# Agronomic biofortification in crops with Zn: the zinc-iron interaction dilemma

**DOI:** 10.3389/fpls.2026.1780147

**Published:** 2026-04-13

**Authors:** Sovan Debnath, Susmit Saha, Samrat Adhikary, Sudipa Mal, Mahasweta Chakraborty, Sushil Kumar, Asha Ram, Rubina Khanam, Deblina Ghosh, Bishnuprasad Dash, Dhaneshwar Padhan, Siddhartha Mukherjee, Ashok Yadav, Biplab, Tufleuddin Biswas, Suddhasuchi Das, Majji Kiranmai Reddy, Anamika Sinha, Kaushik Batabyal, Dibyendu Sarkar, Biswapati Mandal

**Affiliations:** 1 Department of Agricultural Chemistry and Soil Science, Faculty of Agriculture, Bidhan Chandra Krishi Viswavidyalaya, Mohanpur, West Bengal, India; 2 Indian Council of Agricultural Research (ICAR)-Central Agroforestry Research Institute, Jhansi, Uttar Pradesh, India; 3 College of Agriculture, Bidhan Chandra Krishi Viswavidyalaya, Burdwan Sadar, West Bengal, India; 4 Dhaanya Ganga Krishi Vigyan Kendra, Ramakrishna Mission Vivekananda Educational and Research Institute, Murshidabad, West Bengal, India; 5 School of Agriculture, Sanskriti University, Mathura, Utter Pradesh, India; 6 Division of System Research and Engineering, Indian Council of Agricultural Research (ICAR)-Research Complex for North Eastern Hill Region, Umiam, Meghalaya, India; 7 Indian Council of Agricultural Research (ICAR)-Central Rice Research Institute, Cuttack, Odisha, India; 8 Faculty of Agriculture, Jyodh Ishwar Singh (JIS) University, Kolkata, West Bengal, India; 9 Department of Soil Science, M.S. Swaminathan School of Agriculture, Centurion University of Technology and Management, Paralakhemundi, Odisha, India; 10 Central Silk Board (CSB)-Central Sericultural Research and Training Institute, Mysuru, Karnataka, India; 11 Division of Agriculture, Faculty Centre of Agriculture, Rural and Tribal Development, Ramakrishna Mission Vivekananda Educational and Research Institute, Ranchi, Jharkhand, India; 12 Symbiosis Statistical Institute, Symbiosis International (Deemed University), Pune, Maharastra, India; 13 Malda Krishi Vigyan Kendra, Uttar Banga Krishi Viswavidyalaya, Malda, West Bengal, India; 14 Department of Environmental Science, School of Sciences, Gandhi Institute of Technology and Management, Visakhapatnam, Andhra Pradesh, India

**Keywords:** biofortification, cereal, green revolution, micronutrient malnutrition, nutrient interactions, Zn-enriched fertilizer

## Abstract

Agronomic biofortification of staple food crops with external supply of micronutrients offers a viable solution to alleviate the plant diet-based micronutrient deficiency, without any yield trade-off. Zinc (Zn) has been one of the key targeted micronutrients in the biofortification program of Sub-Saharan Africa, Middle East and South-East Asia. Agronomic Zn biofortification, while promising for addressing Zn deficiencies in humans, raises valid concerns regarding its potential impact on grain iron (Fe) content. This is because Zn and Fe can interact at multiple levels, within soil, plant root, shoot, leaves and grains during transport and translocation. Emerging evidences suggest that biofortifying crops with Zn may trigger systemic responses that alter Fe homeostasis, potentially lowering Fe accumulation (antagonistic) in edible tissues of staples. Nevertheless, there is a lack of clarity in the field studies on the effects of Zn biofortification on Fe levels. This review article aims to synthesize a robust narrative on Zn-Fe interactions in biofortified crops and possible causes of negative interaction within plant body. Though, the additive or synergistic interaction between these two essential micronutrients is advantageous in a Zn biofortification program; but such interaction may be highly local stress specific. On the contrary, any loss of Fe during the process is undesirable, as the nutritional significance of Fe is comparable to that of Zn in human health. Yet, unlike other nutrient interactions (e.g., phosphorus (P) × Zn, nitrogen (N) × Zn), their interaction (Zn-Fe) is much less examined. But now, amid reports on unwitting depleting trend in grain micronutrients content in cereals, observed across the globe over the past 70 years, Zn - Fe interaction should apparently be given importance. In this review, we also highlighted on some possible interventions, agronomic or genetic or integrated approaches, to discard such negative interactions between Zn and Fe for future biofortification program. Addressing this trade-off (Zn-Fe interactions in crops) is the key to ensure that biofortification strategies do not inadvertently raise other micronutrient deficiencies. A focus on feasible management practices to curb such negative interaction between Zn and Fe will determine the success of agronomic Zn biofortification.

## Introduction

Plants and humans rely highly on adequate Zn and Fe supply for critical physiological processes, health prosperity and ability to reproduce (Olsen and Palmgren, 2014; Huang et al., 2020). In plants, Zn is involved in enzyme activation, protein synthesis, pollen development and chlorophyll production (Clemens, 2022; Stanton et al., 2022). Likewise, Fe is crucial for chlorophyll synthesis, N metabolism, and plant defense (Rengel et al., 2023; Vélez-Bermúdez and Schmidt, 2023). In humans, Zn serves as a cofactor in as many as 300 enzymes (Alloway, 2008); while, Fe is a fundamental constituent of hemoglobin, an indispensable oxygen carrier (Briguglio et al., 2020). Both nutrients remain critical in cognitive development of the children until adolescence (Garcia-Oliveira et al., 2018).

**Figure 1:**
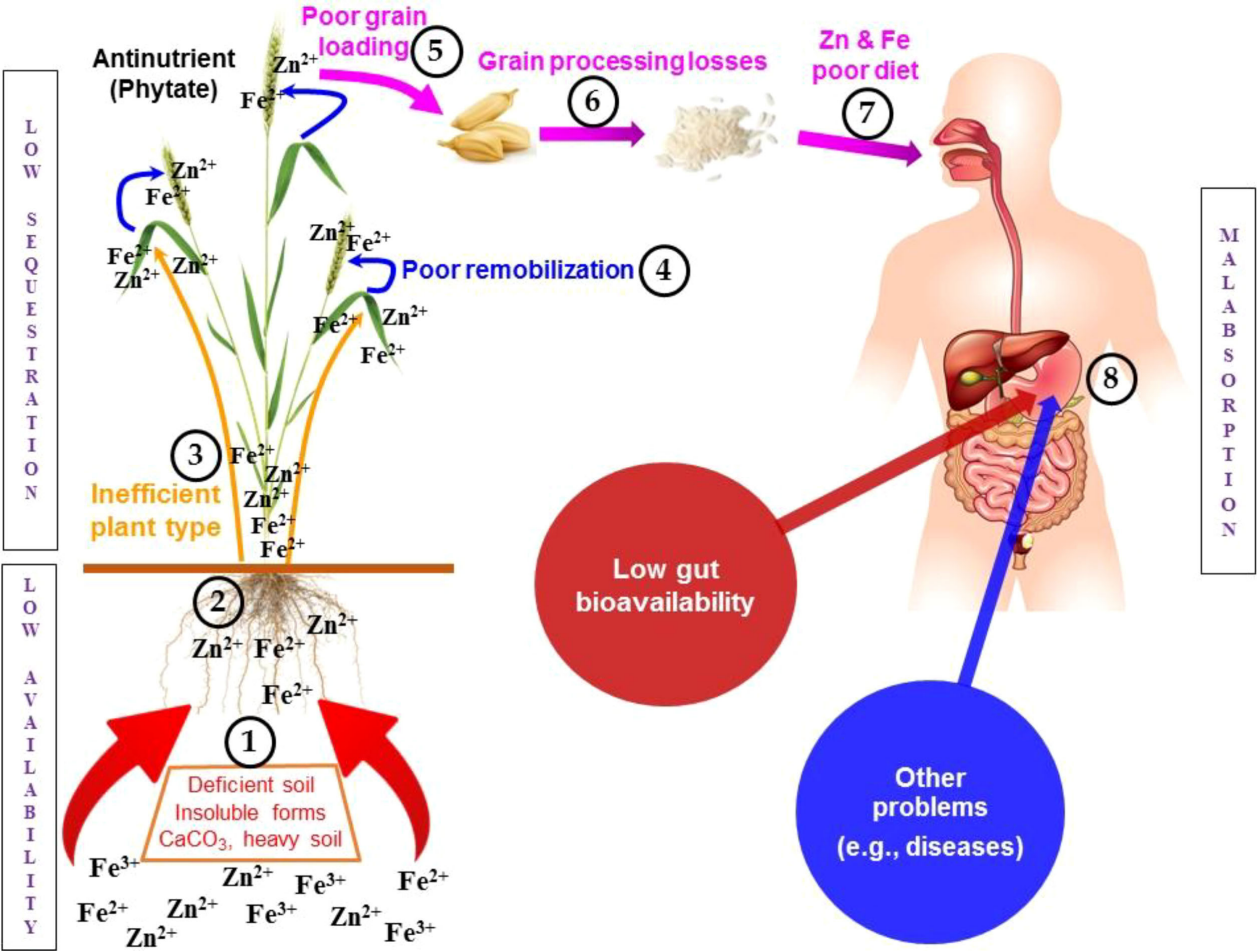
Key reasons of Zn and Fe deficiency in soil-plant-human continuum contributing to the global malnutrition in humans (1). Adversities of Zn and Fe phyto-availability in soils that includes inherently deficient soils, calcareousness/alkalinity, high pH/clay content, transformation to insoluble forms (precipitation, occlusion etc.), high soil organic matter, low soil nitrogen availability (2); Nutrient interactions at the root level acquisition either for root epidermis-secreted phytosiderophores or for carrier proteins that hinders Zn and Fe acquisition by the roots from soil (3); Antagonism between Zn and Fe during the long-distance root to shoot translocation owing to their competition for specific carrier proteins (ZIP, YSL family proteins) that interferes with the chelation process and xylem unloading (4); Possible Zn and Fe antagonism during remobilization from leaves (source) to developing seeds (sink) as the plant progresses towards maturity (5); In the cereal grains, Zn and Fe are highly localized and partitioned to the nutrient-dense embryo (germ) and outer aleurone layer (bran) but lower accumulation in the carbohydrate-dense endosperm (what we actually eat) and co-accumulation of inositol hexaphosphate (phytic acid), a dietary inhibitor with high cation-binding affinity (6); Following harvesting, cereal grain is dehydrated and is then hulled to eliminate the uneatable hull and further milled to remove germ and bran fractions for obtaining white rice/cereal flour that is devoid of Zn and Fe-dense embryo and aleurone layer (7); Cleansing of white rice, cooking with excess water, discarding the gruel, and frying at high temperature leads to further loss of Zn and Fe in the parboiled rice (8); Presence of phytic acid in cereals chelates metal ions such as Zn and Fe to form insoluble phytate that markedly prevents their absorption in the human intestine as well as impairment of absorption due to diseases like inflammatory bowel diseases, Crohn’s disease, Celiac disease, Cystic fibrosis etc., overall leading to Zn and Fe malnutrition.

A widespread soil-plant-human continuum of Zn deficiency is reported on a global scale, compounded by - a) low phyto-availability, b) poor translocation from soil to grain/seed, and c) low bioavailability of the metal in humans (**Figure 1**). For example, reports from the international literature estimate ~50% Zn deficient arable lands in Asia, South Asia and Middle-East (Alloway, 2009). Meanwhile, its insufficiency adversely affects ~1.2/2 billion global populations (17%), with a higher preponderance in sub-Saharan Africa (24%) and South-East Asia (19%) (Bailey et al., 2015). Iron is the fourth most abundant element in the Earth’s crust (Kabata-Pendias, 2000). Inability of this concentrated element to translocate from soil to plant parts causes extensive prevalence of Fe deficiency, affecting ~33% of the global population (Pasricha et al., 2021). Moreover, different edaphic factors like calcareousness, alkaline soil reaction, salinity, sandy texture, poor drainage, and low soil organic matter (SOM) adversely affects the phyto-availability of Zn and Fe (Alloway, 2008; Dapkekar et al., 2018).

Addressing Zn and Fe deficiencies is urgent due to their widespread effects on human health. To improve Zn and Fe phyto-availability from soils, several strategies can be adopted. The possible strategies may be - i) Conjoint foliar application of Zn- and Fe-enriched chemical fertilizers, ii) adjusting soil pH (Kabata-Pendias, 2000; Rengel et al., 2023) and iii) the use of organic amendments to form soluble metal-chelates (Dhaliwal et al., 2021). The regular practice of intercropping, crop rotation, agroforestry and the use of biofertilizers may also smoothen Zn and Fe translocation from soil to plant. Organic farming accompanied with suitable crop rotation may also be an effective option to support Zn and Fe bio-availability in plants (Hou et al., 2025). Zinc and Fe fertilization through soil, foliar, seed priming or in a combination have been proven effective in minimizing their plant deficiencies (Thapa et al., 2021; Thapa et al., 2022), improving grain yield and nutrient content (Mathpal et al., 2015; Saha et al., 2017a; Debnath et al., 2021a). However, the efficacy of the applied fertilizers depends on several soil conditions like good drainage, favorable soil reaction (slightly acidic to neutral), and adequate soil organic matter. Beyond refining grain loading with Zn and Fe, application of such fertilizers substantially reduces the phytate-to-metal molar ratio for an improved nutrient bioavailability (Saha et al., 2017b; Chakraborty et al., 2024). However, under alkaline conditions, these applied fertilizers in soil are literally wasted, contributing to environmental pollution.

The success of Zn-fertilizers (e.g., ZnSO_4_. 7H_2_O, Zn-EDTA) for enhancing grain Zn content (agronomic biofortification) is now globally recognized (Kanwal et al., 2010; Zou et al., 2012; Shivay et al., 2013; Dhaliwal et al., 2019; Liu et al., 2020; Palacio-Márquez et al., 2022), which may warrant its needed supply to the target populations (Alloway, 2009; Tam et al., 2020). The Zn-Fe interaction, through agronomic biofortication of Zn, sometimes has been reported as synergistic (Cakmak et al., 2010) or indifferent, sometimes as negative (Saha et al., 2017b). Calcareous and alkaline soils are generally very low in plant available Zn and Fe. Application of Zn in such soils may boost up Fe absorption through genetic signaling of crops (Cakmak et al., 2010), showing Zn-Fe synergism. But Zn induced Fe depletion (Zn-Fe antagonism on Zn application) in crops is also obvious for the soils with low to marginal Zn but adequate plant available Fe (Habib, 2009; Gopal and Nautiyal, 2012; Saha et al., 2015; Saha et al., 2017b; Niyigaba et al., 2019; Debnath et al., 2021b; Kumar and Dhaliwal, 2022). Thus, absorption of Zn and Fe through plants and their interaction for the long-distance transport essentially depend upon local abiotic stress pattern. Alteration of expressions of different genes, associated with metal translocation in plant body, is obvious in different agro-climatic zones. Therefore, the success of agronomic biofortification does not depend merely on choice of fertilizers and standardization of application protocol. In spite, the success depends upon cumulative consideration of i) proper choice of cultivars for different agro-ecological regions, ii) better understanding of metal mobility within the plant body, iii) targeting particular genes for improved metal transport and iv) probability of interactions among the metals (Zn and Fe), acquired from local abiotic stress and altered genetic signaling, in different crop varieties. This review article insights a good number of experimental outcomes where different Zn-Fe interactions, either positive and indifferent or negative, sustained as a function of genetic expressions depending upon local abiotic stress. Zinc and Fe synergism benefitted the agronomic biofortification program in different regions over the globe. Simultaneously, this is also of particular concern when additional Zn depletes Fe in some region, compromising plant and human nutrition for both metals. This observation prompts a key question: can Zn biofortification be achieved without depleting Fe accumulation in crops? Addressing this trade-off is essential to ensure that the biofortification strategies will not inadvertently exacerbate other micronutrient deficiencies. Zinc induced Fe deficiency may be further manipulated by three possible options. These are – i) prioritization of congenial trait inclusion to new crop mutants through genetic biofortification, ii) proper screening and choice of efficient cultivars for the soils with low Zn but high Fe availability, and iii) conjoint application of Zn and Fe fertilizers on crops to curb the possibility of Zn-Fe antagonism. Earlier review articles on Zn and Fe biofortification highlighted on success stories of such program over the globe. Some other review articles also critically discussed upon molecular mechanisms of metal interactions within the plant body. This review article aims to discuss not only on Zn-Fe negative interactions but also to provide an insight from every possible angle to mitigate such interaction. Therefore, this review highlights on (i) the underlying mechanisms of the Zn-Fe interaction in plants, (ii) the emerging intensification of Zn - Fe antagonism in new-era crop cultivars, and (iii) potential strategy through integrated approaches combining agronomic, genetic and proper screening of varieties to modify Zn-Fe antagonism in crops.

## Strategic measures for curbing Zn and Fe malnutrition and their constraints

In view of global prevalence Zn and Fe deficiencies in humans, effective approaches are adopted through pharmacological supplementation, post-harvest food fortification, dietary diversification, or through food grain biofortification by agronomic or transgenic means (Kumar et al., 2019). However, supplementation programs and social education to revamp choice of foods in diets in vulnerable populations have achieved inadequate success in curbing micronutrient malnutrition (Sazawal et al., 2010), and mostly relies on continued financial incentive and infrastructure (Tam et al., 2020). Recent reports indicate that these micronutrients interact to negatively affect their absorption and/or blood serum level in humans on supplementation (De Brito et al., 2014; Rolf et al., 2021), and thus may have fatal consequences. On the other side, individuals consuming lesser amounts of food, like toddlers, might not possibly attain adequate intake of micronutrient from the fortified foods (World Health Organization, 2006). High costs associated with the fortified processed foods cannot be afforded by the poorer section of the society, who mainly suffers from micronutrient deficiencies.

Global experiments of micronutrient fortification have revealed moderate impact on the growth of children and women (**Table 1**). The studies consistently demonstrated significant reductions in Fe deficiency across settings; however, improvements in Zn deficiency were less consistent, with several studies reported persistent Zn deficiency despite the interventions. The success of fortification programs depends on maintaining a stable market and uninterrupted supply chain, which is often difficult in low-income settings. Moreover, critical monitoring is needed to ensure the fortificants will not interfere with the shelf life, color, taste and quality of consumable food products (Das et al., 2020). Fortified food products distributed at the population level may exclude economically disadvantaged groups. On the other hand, dietary modification needs replacement of plant-source food products by nutrient-dense animal-source ones (e.g. red meat, fish, and eggs) (**Table 2**). Legumes, leafy vegetables, and animal-source foods emerge as comparatively rich micronutrient sources to meet dietary requirements. While these foods provide far greater Zn and Fe than cereals, achieving such diversification in vulnerable populations is often difficult.

**Table 1:** Characteristics, intervention design, and effectiveness of iron (Fe) and zinc (Zn) food fortification programs among children across diverse geographic and socio-economic settings.

Study location	Samples (*n*)	Sample characteristics	Intervention products	Zn/Fe fortification (daily dosage)	Duration of interventions	Retention rate	Compliance	Significant benefit (curbing micronutrient deficiency)	Reference
Botswana	311	Urban	240 ml fruit beverage daily	7.0 mg Fe, 3.75 mg Zn	8 weeks	85%	89%	Yes	(Abrams et al., 2003)
Tanzania	830	Rural	250 ml fruit beverage 5 days per week	5.4 mg Fe, 5.25 mg Zn	6 months	93%	80%	Yes	(Ash et al., 2003)
Bangladesh	1125	Girls, Rural	200 ml fruit beverage 6 days per week	7.0 mg Fe, 7.5 mg Zn	12 months	92%	Not reported	Reduced Fe deficiency, but Zn deficiency sustained	(Hyder et al., 2007)
Northeast Thailand	569	Rural	Seasoning powder added to daily lunch	5.0 mg Fe, 5.0 mg Zn	31 weeks	98%	75%	Reduced Zn deficiency, but Fe deficiency sustained	(Manger et al., 2008)
Vietnam	510	Rural	30 g biscuits, 5 days per week	6.0 mg Fe, 5.6 mg Zn	4 months	91%	95%	Yes	(Nga et al., 2009)
Adelaide, Australia	396	Urban	100 ml fruit flavored soy drink daily	10.0 mg Fe, 5.0 mg Zn	12 months	70%	76%	Reduced Fe deficiency, but Zn deficiency sustained	(NEMO Study Group, 2007)
Jakarta, Indonesia	384	Urban	100 ml fruit flavored soy drink and three biscuits, 6 days per week	11.0 mg Fe, 5.0 mg Zn	12 months	96%	86%	Reduced Fe deficiency, but Zn deficiency sustained	
Hyderabad, India	328	Middle Socio Economic Status	150 ml milk porridge daily	14.0 mg Fe, 2.3 mg Zn	14 months	74%	Not reported	Reduced Fe deficiency, but Zn deficiency sustained	(Sivakumar et al., 2006)

**Table 2:** Reported concentration ranges of zinc (Zn) and iron (Fe) in selected plant- and animal-based foods (on dry weight basis).

Consumable food class	Zn (mg kg^-1^)	Fe (mg kg^-1^)	Reference
1. Cereals			
Rice	13.5-58.4	7.5-24.4	(Welch and Graham, 2004)
Wheat	25.2-53.3	28.8-56.5	
Maize	14.7-24.0	16.4-22.9	
Sorghum	11.2-75.8	11.0-95.4	(Badigannavar et al., 2016)
Barley	20.0-49.7	22.6-36.7	(El-Haramein and Grando, 2008)
2. Legumes			
Chickpea	35.0-60.0	24.0-41.0	(Zia-Ul-Haq et al., 2007)
Common Bean	21.0-54.0	34.0-89.0	(El-Haramein and Grando, 2008)
Pea	16.0-107.0	23.0-105.0	(Grusak and Ismail, 2005)
Soybean	31.5-39.3	38.4-90.6	(Wiersma and Moraghan, 2013)
Lentil	29.7-64.0	26.9-111.2	(Debnath et al., 2022)
3. Roots and tubers			
Cassava	3.0-38.0	6.0-230.0	(Chávez et al., 2005)
Potato	8.0-20.0	9.0-237.0	(Burgos et al., 2007)
4. Vegetables			
Spinach	31.0-387.0	50.0-139.0	(Grusak and Ismail, 2005)
Tomato	18.9-36.2	24.6-67.4	(Debnath et al., 2022)
Brinjal	14.6-19.8	20.6-25.9	(Chakraborty et al., 2022)
5. Fruits			
Banana	13.1-18.2	18.1-23.6	(Chakraborty et al., 2022)
Mango	9.3-28.4	15.1-32.8	(Debnath et al., 2022)
6. Foods from animal origin			
Milk	5.0-6.2	0.9-1.1	(Singh et al., 2019)
Chicken	35.8-41.4	63.1-87.8	(Korish and Attia, 2020)
Eggs	58.6-68.3	11.8-12.5	
Fish	10.1-91.9	29.4-370.0	(Molla et al., 2021)

Agronomic biofortification through micronutrient fertilization is an inexpensive, environmentally benign, and sustainable approach to circumvent these problems by increasing their content in staples (Saha et al., 2017b; Saha et al., 2017a; Chakraborty et al., 2024), particularly cereals without yield trade-off. As per an estimate, agronomic biofortification of wheat and rice with Zn could solely save as many as 0.20 million lives in India (Tarafdar et al., 2014). This strategy can effectively complement the other approaches through delivering micronutrient rich food to the undernourished mass (Andersson, 2017). It is important to highlight that agronomic biofortification with Zn has been proved to be a successful endeavor as compared to Fe biofortification programs (Alloway, 2008; Zou et al., 2012). On a global scale, agronomic biofortification through Zn fertilization resulted in enhanced yield as well as higher Zn content (10-220% increase over no Zn) in the edible parts of food crops, and improved overall plant growth depending on crop species, genotype, soil type, and Zn application methods (**Table 3**). Zinc fertilizers can be applied in different methods including seed priming, root dipping, soil addition, foliar spray, and/or a combination of those. However, combined soil and foliar strategies frequently produce superior Zn enrichment compared to single-mode applications (Pahlavan-Rad and Pessarakli, 2009; Abdoli et al., 2016; Kopittke et al., 2019). The findings support agronomic biofortification as a practical, scalable strategy for improving dietary Zn intake while maintaining or enhancing crop productivity.

**Table 3:** Agronomic zinc (Zn) biofortification strategies showing percentage increases in crop yield and Zn concentration in edible plant parts under different fertilizer sources, application methods, and doses.

Crop	Country	Zn fertilizer used	Zn fertilization		Increase in yield (%)	Increase in Zn content (%)	Reference
			Method	Dose			
Durum wheat	India	ZnSO_4_.7H_2_O	Foliar	0.5%	5	107	(Dhaliwal et al., 2019)
Wheat	Kazakhstan	ZnSO_4_.7H_2_O	Foliar	0.5%	2	216	(Zou et al., 2012)
Wheat	Mexico				4	105	
Wheat	Zambia				Nd	87	
Wheat	India	ZnSO_4_.7H_2_O	Soil + foliar	23 kg ha^-1^ + 0.5%	13	68	(Chakraborty et al., 2024)
Triticale	India	ZnSO_4_.7H_2_O	Foliar	0.5%	4	111	(Dhaliwal et al., 2019)
Rice	Thailand	ZnSO_4_.7H_2_O	Soil + foliar	50 kg ha^-1^ + 0.5%	1	90	(Phattarakul et al., 2012)
Rice	Lao PDR				6	99	
Rice	China	ZnSO_4_.7H_2_O	Foliar	0.5%	7	42	(Yuan et al., 2013)
Rice	Pakistan	ZnSO_4_.7H_2_O	Soil	75 kg ha^-1^	Nd	177	(Amanullah et al., 2020)
Rice	India	ZnSO_4_.7H_2_O	Soil + foliar	20 kg ha^-1^ + 0.5%	Nd	95	(Saha et al., 2020)
Pearl millet	India	ZnO nanofertilizer	Foliar	10 mg L^-1^	38	10	(Tarafdar et al., 2014)
Oat	India	ZnO	Soil	6.25 kg ha^-1^	12	26	(Shivay et al., 2013)
Maize	Pakistan	ZnSO_4_.7H_2_O	Soil	18 kg ha^-1^	21	40	(Kanwal et al., 2010)
Maize	China	ZnSO_4_.7H_2_O	Soil	150 kg ha^-1^	17	75	(Liu et al., 2020)
Maize	India	ZnSO_4_.7H_2_O	Soil + foliar	25 kg ha^-1^ + 0.5%	Nd	36	(Saha et al., 2015)
Cowpea	India	ZnSO_4_.7H_2_O	Soil + foliar	16 kg ha^-1^ + 0.3%	44	23	(Kumar and Dhaliwal, 2022)
Common bean	Mexico	ZnNO_3_	Foliar	50 mg L^-1^	19	92	(Palacio-Márquez et al., 2022)
Potato	Ecuador	Zn-EDTA	Foliar	0.16%	8	131	(Kromann et al., 2017)
Onion	Spain	Zn-aminolignosulfonate	Soil	448 kg ha^-1^	46	212	(Almendros et al., 2015)

Although external supply enhances Zn content of the grains, but the use efficiency of applied Zn fertilizers barely surpasses 2% of the added amount (Alloway, 2008; Debnath et al., 2015; Saha et al., 2017a), and thus may have environmental repercussions. Additionally, Zn-containing ore minerals are limited since its presence in traces in the lithosphere (80 mg kg^-1^) (Mertens and Smolders, 2013), however mined Zn remained the principal source of Zn (60%) used worldwide (Montalvo et al., 2016). Thus, developing novel fertilizer formulations with better use efficiency (Tarafdar et al., 2014; Subbaiah et al., 2016; Dapkekar et al., 2018) or modifying Zn addition protocol to improve translocation of enhanced quantities of Zn to the grains and/or edible parts (Saha et al., 2017a) remains need of the hour and may help to enhance use efficiency of the applied fertilizers.

### Zn × Fe interaction: the inside story

**Figure 2:**
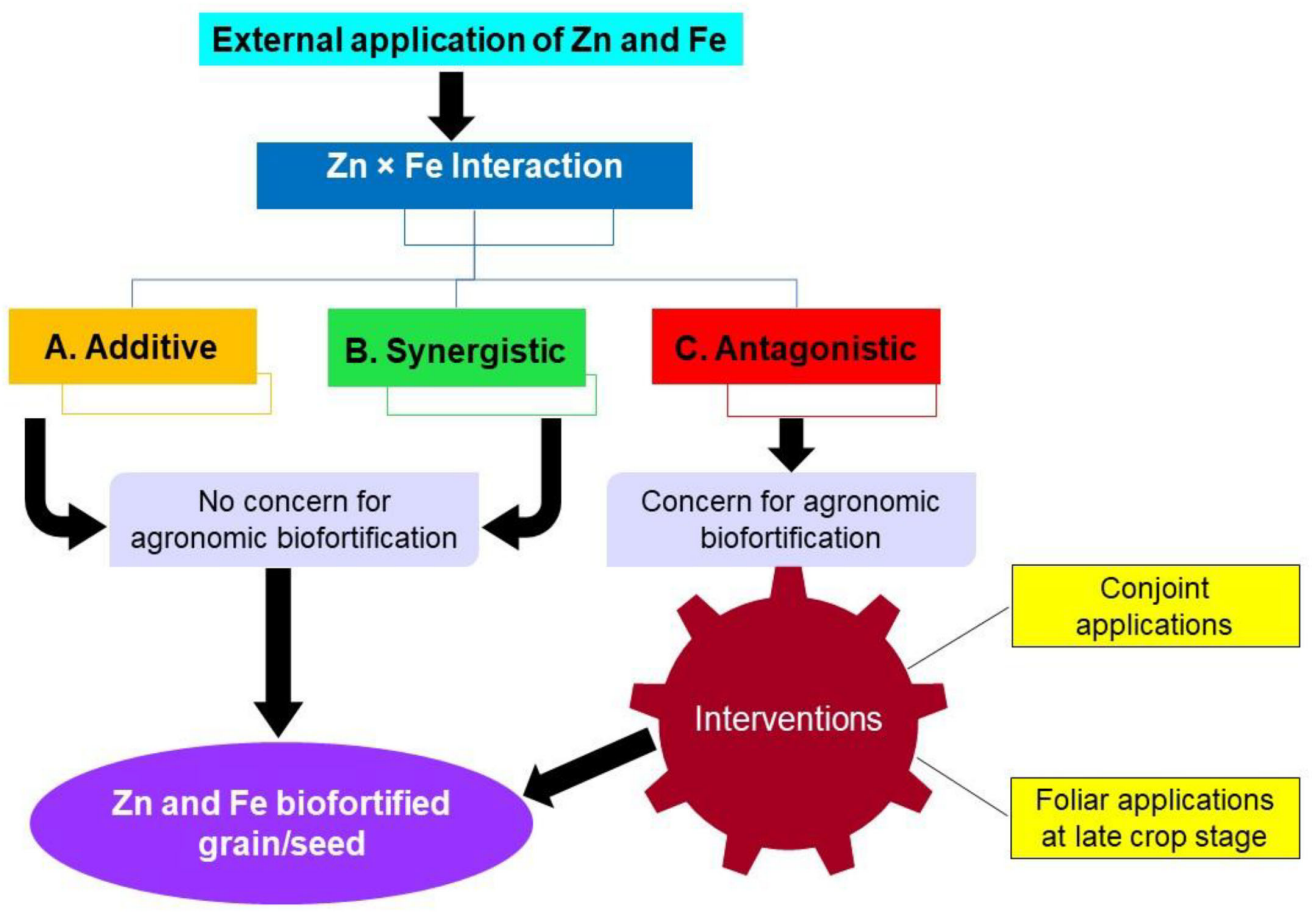
Typical relationships between Zn and Fe in plants in lieu with agronomic biofortification with Zn and Fe fertilizers. Under scenario A and B, little constraints is experienced by the agronomists for a simultaneous biofortification of grain Zn and Fe. Under scenario C, simultaneous biofortification of grain Zn and Fe is severely constrained by their antagonism and requires agronomic interventions including conjoint applications and foliar applications of Zn and Fe fertilizers at late crop growth stage, which ultimately leads to Zn and Fe biofortified grain/seed development.

Irrespective of the method used, extra Zn in the food grains (Food fortication of Zn) added through biofortification can only be a feasible intervention to alleviate its widespread deficiency in the vulnerable populations. However, external supply of Zn may interact with other nutrients including Fe, thereby impacting their absorption and metabolism. Factually, the relationship between Zn and Fe in plants remained elusive so far: often antagonistic (Hamlin and Barker, 2008; Debnath et al., 2021a), and seldom synergistic (Hadi et al., 2015; Hocaoğlu et al., 2020), while sometimes not so prominent (Chakraborty et al., 2022) based on the nature of soil (**Figure 2**). Iron and Zn interaction, and Fe metabolism in plants is closely linked to Zn supply due to shared uptake through the same transport pathways (Narwal and Malik, 2011). Thus, Zn deficiency may possibly accentuate Fe uptake to toxic levels in some conditions. This interaction appears to be a conundrum as those observed between P and Zn in plants; yet it is less studied (Kabata-Pendias, 2000).

Zinc deficiency enhances metal concentrations (such as Fe) in the shoots of both strategy I (non-grass) and strategy II (grass) plants. This is most likely due to rhizosphere acidification mechanisms and release of reducing agents and/or phyto-siderophores. Under Fe deficiency conditions, Zn uptake by plants and Zn concentration in their shoots increased significantly in both grasses and non-grasses (Römheld et al., 1982; Zhang et al., 1991). An experiment on Indian mustard (*Brassica juncea*) showed the inhibitory effect of increasing the level of Fe in the solution on the concentration of Zn in the shoots and roots (Hamlin and Barker, 2008). Some recent studies have highlighted the Zn - Fe interaction during the process of Zn biofortification in crops by agronomic means through application of Zn fertilizers. For example, Shivay et al. (2015) reported that Zn application to the soil significantly increased the concentration of Fe in rice grains. In some cases, foliar Zn-EDTA application significantly increased Fe concentration in grains (Pahlavan-Rad and Pessarakli, 2009), while soil application does not (Pahlavan-Rad and Pessarakli, 2009; Behera et al., 2015). EDTA acts as a ligand for many metal transporters and this may explain the positive effect of Zn application on Fe uptake and sink transport (Kabata-Pendias, 2000). However, these mechanisms can be disrupted in the presence of high level of Fe or Zn.

**Figure 3:**
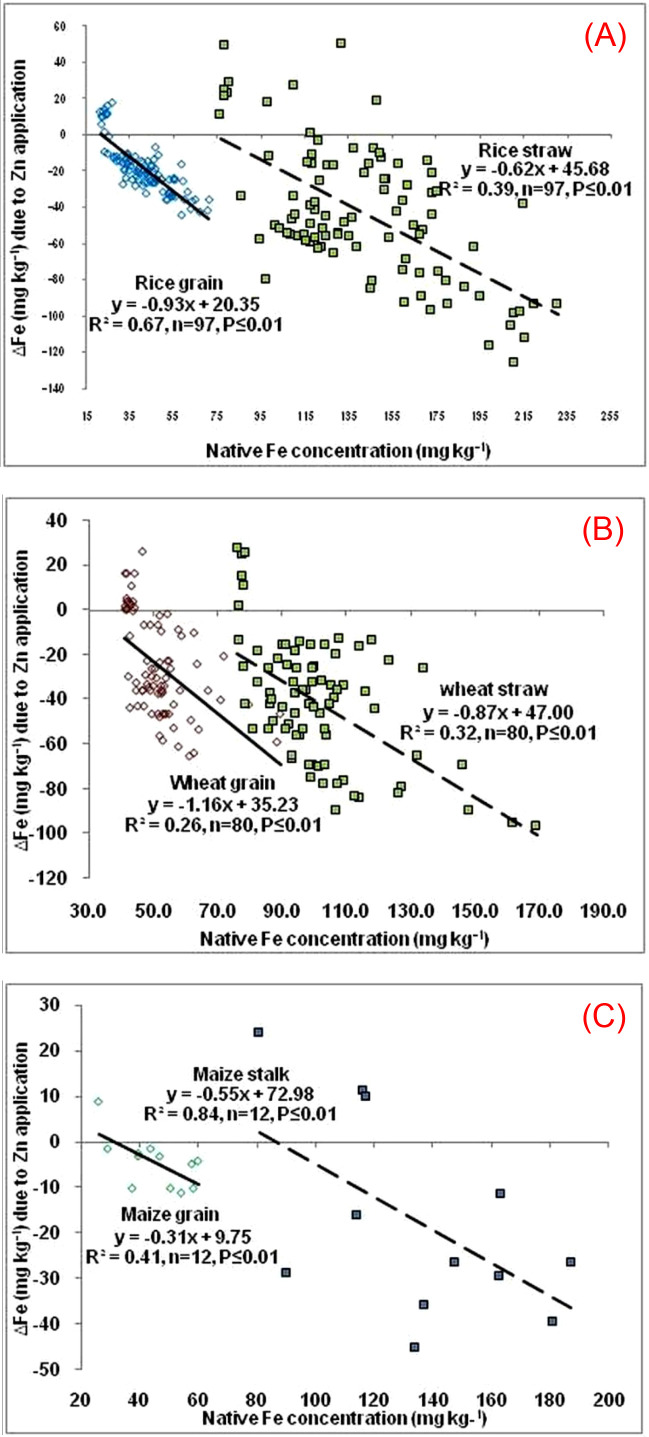
Relationship between the changes in Fe concentration (ΔFe) in the harvested grains and straws/stalks of different cultivars of the cereals due to Zn application and native Fe concentration. **(A)** Rice; **(B)** Wheat; **(C)** Maize (Adapted from Saha et al. (2015).

Synergistic interaction between these two essential micronutrients is an added advantage in the Zn biofortification program. On the contrary, depletion in Fe concentration, if occurs, during Zn biofortification in crops cannot be compensated since Fe is equally important as Zn in human nutrition. However, a number of researchers have reported a significant decrease in Fe concentration with Zn fertilization in different plant parts of rice, wheat, and maize (Gopal and Nautiyal, 2012; Saha et al., 2015; Saha et al., 2020). Such possibility may arise due to either i) dilution effect for higher biomass yield or ii) antagonism between the two metals (**Table 4**). These studies reported that Zn application was consistently associated with a decline in grain Fe concentration, with reductions ranging from ~4% to over 50%, indicating a potential antagonistic interaction between Zn and Fe accumulation in grains. Furthermore, Saha et al. (2015) observed that the antagonism was more acute in rice grains loaded with more than 25 mg kg^-1^ Fe, maize and wheat grains with more than 40 mg kg^-1^ Fe showing the similar trend (**Figure 3**). In another investigation, a greater reduction in Fe in rice grains on combined application of Zn through soil and foliar (17.4%) than one soil application alone (9.4%) (Saha et al., 2020). A similar trend was again observed in cowpea (Kumar and Dhaliwal, 2022). Zn-induced depletion in grain Fe was also noticed when wheat plants were supplied with foliar Zn (Jalal et al., 2020).

**Table 4:** Effect of agronomic zinc (Zn) fertilization on grain Zn concentration and associated changes in iron (Fe) concentration (mg kg^−1^) across multiple crops and cultivar types under combined soil and foliar application strategies.

Crop	Type of cultivars	−Zn		+Zn		% change	
		Zn	Fe	Zn	Fe	Zn	Fe
Rice^§^	Local (n = 47)	16.1	42.7	22.3	23.1	38.1	-45.9
	High yielding (n = 30)	29.1	36.6	40.7	19.9	39.9	-45.6
	Hybrid (n = 20)	20.7	35.2	31.9	26.1	54.1	-25.9
Rice^§^	Local (n = 2)	29.0	67.0	57.7	56.4	98.8	-15.7
	High yielding (n = 17)	29.3	56.4	56.7	47.9	93.3	-15.1
	Hybrid (n = 3)	25.9	58.0	61.8	43.9	138.3	-24.4
	Aromatic (n = 4)	32.1	72.0	55.0	56.2	71.8	-22.0
Wheat^§^	Early sown (n = 40)	25.0	57.7	36.1	27.7	44.4	-51.9
	Late sown (n = 40)	25.9	55.9	37.4	40.4	44.4	-27.7
Wheat^†^	High-yielding (n = 10)	28.7	40.0	77.2	32.5	168.8	-18.8
Wheat^‡^	High-yielding (n = 1)	23.2	54.8	35.6	44.5	53.4	-19.0
Maize^§^	Hybrid (n = 12)	27.9	45.1	37.9	40.6	35.8	-9.9
Maize^Ψ^	High-yielding (n = 1)	22.0	22.7	34.0	18.4	54.5	-18.9
Cowpea^$^	Dual-purpose (n = 1)	31.3	49.2	38.4	47.3	22.7	-4.0

Values within parentheses reflect number of cultivars tested.

−Zn = No Zn.

**^§^** (Gopal and Nautiyal, 2012): +Zn = Soil Zn (basal at 5.0 kg Zn ha^-1^) + two foliar spray of Zn (0.5% as ZnSO_4_.7H_2_O) at maximum tillering/6-8 leaf and flowering/silking stage.

^§^ (Behera et al., 2015): +Zn = Soil Zn (basal at 4.0 kg Zn ha^-1^) + two foliar spray of Zn (0.5% as ZnSO_4_.7H_2_O) at pre-flowering and grain forming stage.

^†^ (Habib, 2009): +Zn = Soil Zn (basal at 20 mg Zn kg soil^-1^) + two foliar spray of Zn (0.5% as ZnSO_4_.7H_2_O) at pre-flowering and 7 days after flowering stage.

^‡^ (Chakraborty et al., 2024): +Zn = Soil Zn (basal at 4.5 kg Zn ha^-1^) + one foliar spray of Zn (0.5% as ZnSO_4_.7H_2_O) at flowering stage.

**^Ψ^** (Guo et al., 2020): +Zn = Soil Zn (basal at 75.0 kg Zn ha^-1^) + two foliar spray of Zn (0.05% as ZnSO_4_. 7H_2_O) at visible collar of 6^th^ leaf and visible last branch of the tassel.

^$^ (Niyigaba et al., 2019): +Zn = Soil Zn (basal at 16.0 kg ZnSO_4_. 7H_2_0.H_2_O ha^-1^) + two foliar spray of Zn (0.3% as ZnSO_4_. 7H_2_0) at early bloom and 1 week after full bloom stage.

The transport of Zn and Fe from soil to seed/grain must pass through many paths in coordination with complex physiological steps. Nicotianamine (NA), a low molecular weight amino acid, is believed to act as a chelate to bind Zn and Fe for long distance transport within the plant body (Clemens et al., 2013). Such long-distance transport involves; firstly, acquisition from soil through the root, and secondly, long-distance transfer from root to shoot, and eventually re-allocation from source (leaves) to the sink (developing seeds/grains) (Carvalho and Vasconcelos, 2013; Garcia-Oliveira et al., 2018), (**Figure 1**). Zinc and Fe antagonism acts on the first two physiological processes. It is well established that Zn competes with Fe at the root level acquisition either for root epidermis-secreted phytosiderophores (von Wirén et al., 1996), or for common transport carriers (Korshunova et al., 1999; Sperotto et al., 2012). Under the condition of excess supply of Zn, the translocation of Fe in plants may be downregulated through over-expression of IRT-1 (Iron Regulated Transporter) (Fukao et al., 2011; Shanmugam et al., 2011). FIT-binding proteins (FBPs) are found to regulate Zn and Fe homeostasis in *Arabidopsis thaliana* (Chen et al., 2018). FBPs can suppress the expression of Nicotianamine synthase genes (NAS) genes in plants to tolerate excess metal conditions (Chen et al., 2018). NAS genes, particularly NAS 1, NAS 2 and NAS 4 show expressions in plant roots (Klatte et al., 2009; Schuler and Bauer, 2011). Therefore, FIT binding proteins (FBPs) may interfere with the expressions of NAS 1, NAS 2 and NAS 4 genes in roots under Zn excess condition, resulting in a strong Zn-Fe competition for root to shoot transport. Moreover, a strong antagonism between Zn and Fe owing to their competition for specific carrier proteins (ZIP, YSL family proteins) during long-distance root to shoot translocation has also been reported. Such a negative interaction interferes with the metal-chelation during their translocation (Kabata-Pendias, 2000; Palmgren et al., 2008) with an inhibitory competition during xylem unloading (Alloway, 2008). Agronomic bioforification with Zn could, therefore, down-regulate Fe uptake or its movement to the developing grain. Conversely, the intensity of antagonism between these two micronutrients comes down during their remobilization from leaves (source) to developing seeds (sink) (Uauy et al., 2006; Saha et al., 2017a). Translocation of Zn and Fe from leaves to grains is controlled by phloem. Genes, YSL 1 and YSL 3 (Yellow Stripe Like 1 and 3) are found to regulate the phloem loading of Zn-NA (Zinc-nicotianamine) chelated complex (Clemens et al., 2013). Vacuolar Iron Transporters, VIT 1 and VIT 2, have been found to be involved in both Zn and Fe transport from flag leaf to developing grain in rice (Zhang et al., 2012). Interestingly, it has been found that Fe is associated with 2’ -Deoxymugineic acid (DMA), instead of NA, in rice phloem (Nishiyama et al., 2012). In wheat, NAC (nascent polypeptide associated complex) domain transcription factor, Grain protein Content 1 (GPC 1) exhibits major role in leaf senescence with Zn and Fe loading in grains (Uauy et al., 2006). It may be presumed that the competition of Zn and Fe for specific binding sites, as mediated by over or under-expressions of different genes, primarily exist during long distance root to shoot transport of these metals. During the reallocation of stored Zn and Fe from leaves to developing grains, no further antagonism occurs, may be due to i) an altered availability low molecular weight organic chelates (NA, DMA) for reduced proportion of metals in leaves, as compared to roots, and ii) Non-existence of FBP and NAS like negative interference in phloem channel.

Genetic interaction that regulates the Zn-Fe relationship within plant body is the prime object needs to be explored for the success of metal biofortification process. FIT binding proteins (FBP) are important to restrict the metal overloading in plants, thus assuring metal homeostasis (Chen et al., 2018). Iron Regulated Transporter (IRT 1), having low substrate specificity, may encourage transport of heavy metals like Cd and Co along with Fe and Zn (Korshunova et al., 1999). In metal excess conditions, a negative interaction between FBP and NAS may be operative in plants to minimize the success of agronomic biofortification of Zn. Genetic biofortification or genetic engineering of plants, therefore, plays a crucial role to overcome such dilemma. Eventually, for developing efficient cultivars, the primary targets should be i) increasing Zn and Fe chelators (NA like organic chelates) through overexpression of NAS genes, ii) implanting more substrate specific metal binding carriers, iii) lowering phytic acid, an antinutrient that restricts metal bioavailability level in new mutants (Stanton et al., 2022) and iv) improving the expressions of genes like MTP 1 (Microsomal triglyceride transfer protein) or HMA 1 (Heavy Metal ATPase) or GPC 1 in new mutant to ensure significant loading of Zn and Fe in seed endosperm.

### Intensification of Zn × Fe antagonism in modern-bred cereals: case studies

During the ‘green revolution’, insertion of a gibberellin-insensitive dwarfing-gene in rice (*sd 1* gene) and wheat (*Rht* gene) ushered a massive enhancement in the productivity of these two staples, as compared to the traditional varieties or landraces. It is quite implausible to expect that these genes would have a pleotrophic effect on grain mineral loading. For example, the inadvertent loss of desirable traits from the domesticated gene pool was substantiated by the identification of an ancestral wild emmer wheat gene (*TaNAM-B1* gene) that encodes a transcription factor, which is not functional in modern wheat cultivars (Uauy et al., 2006). This wild gene is known to control leaf senescence that enhances Fe, Zn, and protein levels. As a fallout of those above phenomena, the modern high-yielding cereal cultivars had inadvertently contributed to decreased micronutrient content in grains in many parts around the world since past more than 70 y, as compared to the traditional crop varieties/landraces (Zhao et al., 2009; Amiri et al., 2015; Hocaoğlu et al., 2020; Debnath et al., 2023; Chakraborty et al., 2024) (**Table 5**). Traditional landraces, particularly in wheat and rice, generally exhibit higher Zn (~13–84 mg kg^-1^) and Fe (~1.0–55 mg kg^-1^) concentrations compared to many modern high-yielding (non-biofortified) cultivars; while, biofortified cultivars demonstrate substantial enhancement of Zn (~16–65 mg kg^-1^) and Fe (~10–73 mg kg^-1^) levels. For example, a study in China by Guo et al. (2020) evaluated 24 maize cultivars, bred and released between 1930-2010, for a temporal change in grain yield and mineral concentrations and found that new-era cultivars (>1963) showed an increase in grain yield ~25.5%; while, a decrease in Cu (0.009 mg kg^-1^ y^-1^), Mn (0.026 mg kg^-1^ y^-1^), and Zn (0.064 mg kg^-1^ y^-1^) concentrations over the old-era cultivars in past 80 y. A recent study by Mayer et al. (2022) with 29 commonly consumed fruits and vegetables of United Kingdom showed a significant depletions in concentrations of Fe (50%), Cu (49%), Na (52%), and Mg (10%) in the overall 80-y-period (1940–2019).While evaluating Australian reference food composition databases between 1991 and 2022, Rangan et al. (2023) observed a temporal decrease of Fe and calcium (Ca) content of 7 cereal grains to the extent of 13.0% and 22.0%, respectively. These reductions have been accompanied by not only a ‘genetic dilution effect’ but a shift in agronomic practices largely from organic practices (pre-historic) to industrial practices (post green revolution), changes in phyto-availability of Zn and Fe in soils with the introduction of nutrient-mining high-yielding cultivars, and elevated CO_2_ in the atmosphere.

**Table 5:** Comparative zinc (Zn) and iron (Fe) concentrations (mg kg^-1^) in traditional landraces, modern high-yielding (non-biofortified), and biofortified cultivars of major crops.

Crop	Cultivar/variety	Zn (mg kg^-1^)	Fe (mg kg^-1^)	Country	Reference
	1. Traditional/landraces				
Wheat	C 306	50–65	40–55	India	(Genc et al., 2010)
	Bansi	40–55	30–40	India	(Rao et al., 2013)
	Kharchia 65	45–60	35–50	India	(Singh et al., 2014)
Rice	Kalanamak	30–40	20–30	India	(Shobha et al., 2006)
	Kalajeera	28–35	22–30	India	(Prasad et al., 2010)
	Kalabath	26.8-38.6	20.7–39.2	India	(Anandan et al., 2011)
	Njavara	35–45	25–35	India	(Shetty et al., 2012)
	Kasturi	45.7	15.0	India	(Anuradha et al., 2012)
	CSR 10	84.0	28.5	India	(Kumar et al., 2012)
	Todal	13.3	0.8	India	(Verma and Srivastav, 2017)
Rice (Aromatic)	Gopal Bhog	17.0	31.5	India	
	2. Modern high-yielding (non-biofortified)				
Wheat	HD 2967	25–35	20–30	India	(CIMMYT, 2015)
	WH 1105	28–36	22–32	India	(Gupta et al., 2015)
	PBW 343	30–40	25–35	India	(Singh et al., 2016)
Rice	MTU 1010	20–25	15–20	India	(ICAR, 2013)
	IR 64	15–20	10–15	Philippines	(ICAR-Directorate of Rice Research, 2016)
	Swarna	22–28	16–22	India	(Pradhan et al., 2015)
	Dzuluorhe	17.5–39.7	7.9–19.9	India	(Naik et al., 2020)
	Mima	17.1–45.6	9.9–17.4	India	
Maize	QPM	10.7-57.8	7.1-58.4	Africa	(Goredema-Matongera et al., 2023)
	3. Modern high-yielding (biofortified)				
Wheat	WB 02	45–60	35–50	India	(Andersson, 2017)
	BHU 1	50–65	40–55	India	(Yadava et al., 2018a)
	HI 8759	42.8	42.1	India	(Yadava et al., 2018b)
	MACS 4028	40.3	46.1	India	
	HPBW 01	48–62	38–52	India	(ICAR, 2019)
	Kabre	20.1-34.7	36.3-44.6	Nepal	(Thapa et al., 2022)
Rice	IR68144	30–38	24–34	Philippines	(IRRI, 2015)
	DRR Dhan 45	25–35	20–30	India	(ICAR-Directorate of Rice Research, 2016)
	BU Aromatic Hybrid Dhan-1	21.8	10.0	Bangladesh	http://dhcrop.bsmrau.net/buaromatic-hybrid-dhan-1
	BU Aromatic Dhan-2	22.0	10.0	Bangladesh	http://dhcrop.bsmrau.net/bu-dhan-2
	Indica	16.0	45.7	Global	(Trijatmiko et al., 2016)
	CR Dhan 310	28–36	22–32	India	(Indian Council of Agricultural Research, 2017)
	Binadhan 20	26.5	20-31	Bangladesh	http://dhcrop.bsmrau.net/binadhan-20
	CENTA A-Nutremas	22.8	6.9	El Salvador	http://marlo.cgiar.org
Pearl millet	HHB 299	41.0	73.0	India	(Yadava et al., 2018b)
Lentil	IPL 220	51.0	73.0	India	
Pomegranate	Solapur Lal	6.4-6.9	56.0-61.0	India	

There remains a possibility that the negative relation between Zn and Fe could have amplified amid unwitting depleting trend in micronutrients concentration of grains during breeding of crops to achieve higher grain yield and disease-pest resistance, causing a massive problem in Zn biofortification program, which mostly remained unheard (Debnath et al., 2021a). Recent studies confirm that a strong antagonism between Fe and Zn subsists in the modern rice cultivars (r= –0.50, *P* < 0.05 (Saha et al., 2017a);, wheat cultivars (r= –0.50, *P* < 0.01 (Debnath et al., 2022); and maize cultivars (r= –0.64, *P* < 0.01 (Saha et al., 2015); released in India. This antagonism is again lately reported in vegetables and fruits (Chakraborty et al., 2022; Debnath et al., 2022; Kumar and Dhaliwal, 2022). Its magnitude, if quantified as decrease in Fe per unit increase in Zn in grains and vice-versa, was found to be greater in wheat varieties than in rice varieties released during 1990s and later than those released during the green revolution in India as observed in a Zn and Fe biofortification experiment (Debnath et al., 2021a) (**Table 6**). Not only antagonism but also responses of the newer released cereal cultivars towards external micronutrient application have also diminished. For example, Debnath et al. (2021a) recently reported decreased response to external Fe and Zn fertilizer to an extent of 13.3 and 9.6, and 8.8 and 10.4% per decade in rice and wheat in last 60 y since beginning of the green revolution in India, respectively. This pattern likely reflects breeding emphasis on yield and agronomic performance during the Green Revolution, potentially narrowing genetic variation for micronutrient uptake and translocation.

**Table 6:** Temporal trends in the responsiveness of rice and wheat cultivars (released from the 1960s to 2010s) to external zinc (Zn) and iron (Fe) fertilization, expressed as mean increases (mg kg^-1^) in grain nutrient concentration.

Decade of release	+ΔZn^§^		+ΔFe^§^	
	Rice	Wheat	Rice	Wheat
1960-69	14.43	14.81	10.52	15.84
1970-79	13.90	11.98	8.88	14.06
1980-89	10.76	11.51	6.00	11.77
1990-99	9.16	8.33	4.73	9.72
2000-09	7.53	7.13	3.52	8.29
2010-19	−	5.61	−	7.52

Values within parentheses reflect number of cultivars tested.

^§^+ΔZn= grain Zn content in Zn treated plants – grain Zn content in Zn untreated plants.

^§^+ΔFe= grain Fe content in Fe treated plants – grain Fe content in Fe untreated plants.

Adapted from (Debnath et al., 2021a).

Values represent means of three different application protocols of Zn and Fe (soil, foliar and soil + foliar).

Are these two essential trace metals becoming stubborn to each other in the newer cereal cultivars? Is such poor relationship vis-à-vis improved yield in cereals exacerbating Zn and Fe malnutrition in humans? The possible reason for this poor relationship with the progress of cultivar development could be attributed to the fact that rate of absorption is often lower than rate of growth in the sink capacity causing a net dilution of these trace metals in grains. Conversely, cultivation on the same piece of land over centuries has caused their widespread deficiency in soils (Shukla et al., 2017; Singh et al., 2023). Recently, Debnath et al. (2021a) reasoned the depletion of Zn and Fe in grains of rice and wheat cultivars over time is either due to their inability to absorb nutrients from the soil or inefficiency to relocate from sources to sinks.

Breaking the dilution effect is indispensable to combat hidden hunger without compromising food security. From agronomic perspective, it requires a transition from simply increasing fertilizer volume to maximizing nutrient use efficiency (NUE) and improving soil health. Precise fertilizer applications (Subbaiah et al., 2016), combining chemical fertilizers with organic sources (Paramesh et al., 2023), tailoring nutrient application to specific crop needs at critical growth stages (Saha et al., 2017a; Debnath et al., 2021a; Yimer and Tarnawa, 2025), and better root foraging ability (Hegde et al., 2007; Liu et al., 2023) ensures nutrients are used effectively, not just in large quantities. From genetic perspective, decoupling of Zn/Fe trait from high yield trait may be helpful so that grain nutrient content remains independent of yield potential. Studies have successfully identified genotypes (e.g., in rice and wheat) that possess both high Zn/Fe concentration and high yield potential (Bhattacharjee et al., 2020; Thapa et al., 2022). For example, in India, several biofortified wheat varieties (e.g. HUW 838, PBW 872, MACS 6768) have recently been released for specific agro-climatic zones, which are high in grain Zn (≥40.7 ppm) and Fe (≥41.2 ppm) contents with excellent yield (≥5.1 t ha^-1^) (Gupta et al., 2023). However, one concern remains about the uncertainty of genetic and environment interaction on the stability of such varietal traits. Recently, Thapa et al. (2022) reported moderate to high heritability for both Zn and Fe in biofortified wheat across diverse environments in Nepal. Thus, more research should focus on wider adaptability of those varieties in varied agro-climatic regions. This will ensure their outreach to the malnourished population on a global scale.

## Overcoming Zn × Fe antagonism for simultaneous biofortification

**Figure 4:**
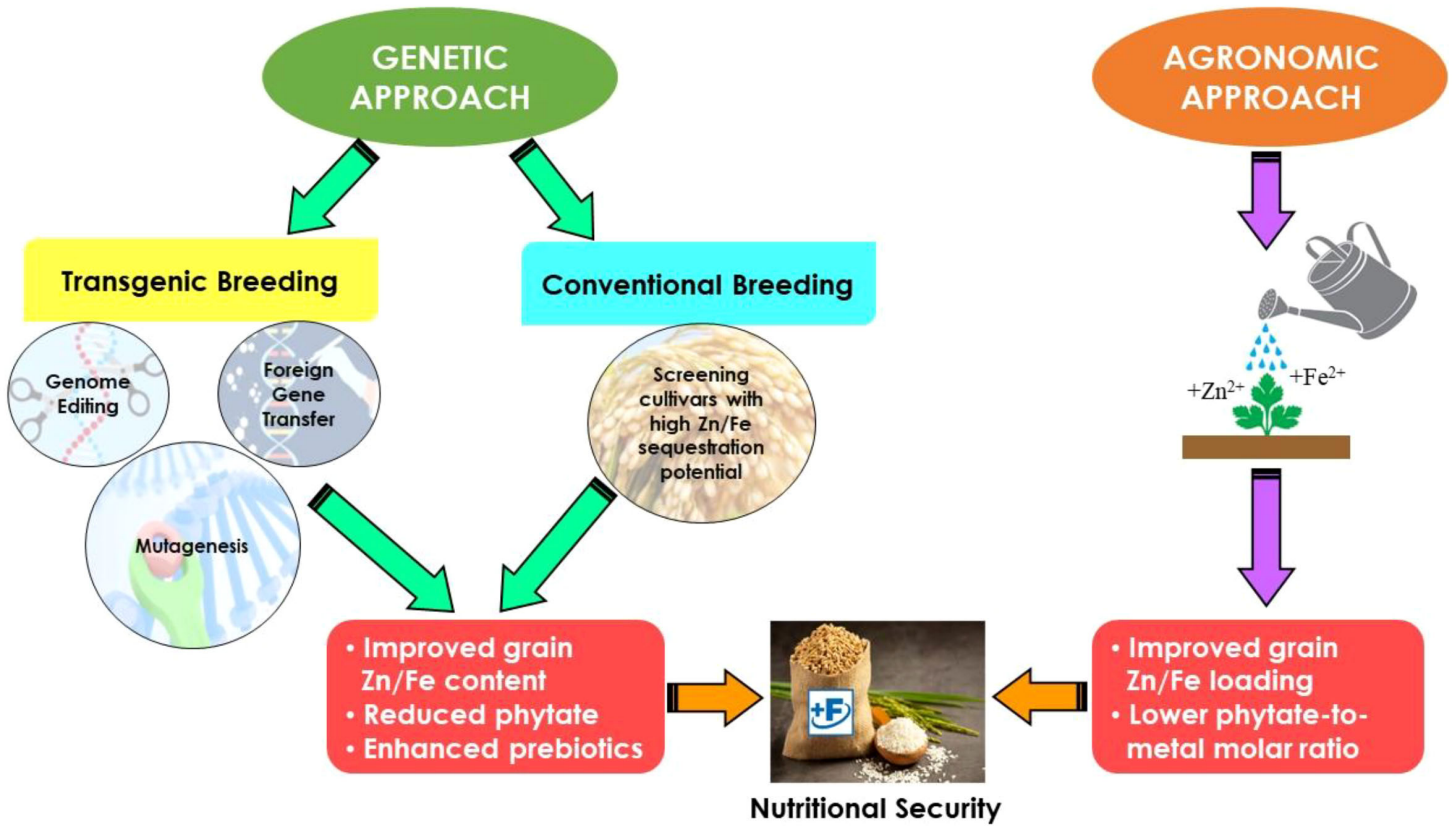
Strategic interventions for improving the contents of Zn and Fe ingrain simultaneously and assuring nutritional security. The biofortification techniques can be categorized into two broad approaches, one which employs agronomic approaches (i.e., use of micronutrient fertilizers) and the other that employs a genetic breeding approach (conventional and transgenic). Conventional breeding approach involves exploring genetic heterogeneity in the natural gene pool to enhance grain mineral contents in the staple food crops, while transgenic breeding approach involves transfer and expression of beneficial genes to the targeted crops from any other plant species, regardless of their evolutionary or taxonomic rank to develop biofortified staple food crops.

Although there is a growing number of studies on the physiology and absorption of individual minerals (Zn, Fe) in plants, there is still no clear understanding of how these minerals interact during the process of root uptake and translocation to vegetative tissues as well as developing seeds (Olsen and Palmgren, 2014; Ricachenevsky et al., 2015). It, therefore, remains one of the obstacles for simultaneous biofortification. Poor understanding of the mechanisms underlying their interplay further complicates the situation. Previous discussions clearly underscore the possible adversities of external Zn application on grain Fe contents. To overcome this bottleneck, there could be two key interventions; firstly a conjoint (Zn + Fe) application and secondly a tailored foliar feeding at a specified crop phenology, at least from agronomic point of view (**Figure 4**). For example, (Habib, 2009) observed a ~1.3-fold higher wheat seed-Fe in a conjoint foliar-fed treatment (Zn and Fe 150.0 g ha^-1^ each) at tillering and heading, as compared to sole foliar-fed Zn. Another study by Saleem et al. (2016) proved an edge of conjoint foliar-fed Zn and Fe (0.1% each) to increase the nutrient contents (107.3% Fe and 122.4% Zn over control) in maize grain over their conjoint soil applications (44.8% Fe and 55.2% Zn over control). Arabhanvi and Hulihalli (2018) further observed that a conjoint soil + foliar-fed Zn and Fe (0.5-1.0% each) was even a better way to enhance Zn (30% increase) and Fe (14% increase) in the grain of sweet corn when compared with a conjoint soil application (10.0 kg ha^-1^ ZnSO_4_. 7H_2_0 and FeSO_4_ each). Recently, Niyigaba et al. (2019) reported an increase by 8-28% Fe and 16-67% Zn in wheat grain with a varied conjoint foliar-feeding (0.26-2.40 kg Zn eq. ha^-1^ and 0.22-2.10 kg Fe eq. ha^-1^), while a 5% decrease in Fe and a 10% decrease in Zn with foliar-fed Zn (3.00 kg Zn eq. ha^-1^) and Fe (2.60 kg Fe eq. ha^-1^) over no Zn and Fe application, respectively.

Can we overcome the antagonistic interaction, if not, through maneuvering their uptake by plant roots? Foliar feeding of Zn and Fe will not undergo the obstacles root feeding usually undergoes. However, postponing foliar feeding from early (vegetative) to late phenological stages (reproductive) could be more effective in enhancing their simultaneous sequestration in cereal grains (Chakraborty et al., 2024). Targeted foliar feeding during their remobilization from source to sink tissues (i.e., at late crop phenology) may also effectively reduce antagonism between Zn and Fe from reaching onto grains possibly due to increased synthesis of abundant Zn-Fe-specific transporters at late crop phenology (Ricachenevsky et al., 2015). For example, Saha et al. (2017a) reported a pronounced antagonistic effect when Zn was applied early at seeding via soil, compared to later foliar feeding at anthesis in wheat. The authors opined that the onset of senescence and degradation of Zn-containing proteins following late-stage application may preferentially enhance the concurrent translocation of Zn and Fe from source to sink. Conversely, some reports suggested that foliar-fed Zn near crop maturity decreased grain Fe concentration and vice versa (Soleimani, 2012; Niyigaba et al., 2019). These contrasting findings underscore the need for further investigation into the effects of foliar-fed Zn during late phenological stages on grain Fe accumulation. Furthermore, the leaf absorption of foliar-fed Zn and Fe is likely modulated by multiple factors, such as type of fertilizer, nutrient content in the spray solution, foliage characters, and timing of application.

Although agronomic biofortification holds potentials and opportunities for enhancing micronutrient contents in staple food crops it also suffers from shortfalls, which are discussed in the preceding sections. Alternatively, the enhanced accumulation of Zn and Fe in staple grains can also be achieved by genetic manipulation of Zn and/or Fe homeostasis-related genes in plants, better known as genetic biofortification (Mhaske, 2019; Das et al., 2020; Senguttuvel et al., 2023). It aims to produce genetically manipulated staple food crops with higher micronutrient levels, reducing levels of dietary inhibitor (e.g. phytic acid), and increasing the levels of substances that promote nutrient absorption (Gregorio et al., 2000). Genetic biofortification can be categorized into two strategies: conventional breeding and transgenic breeding (**Figure 4**). In conventional breeding, superior-quality parent lines with high Zn and Fe content are crossed with recipient lines with other desirable agronomical qualities (e.g. yield, biotic stress tolerance) to develop plants with higher grain Zn and Fe content without a trade-off on grain yield (Van Der Straeten et al., 2020). Transgenic breeding involves transfer and expression of beneficial genes from any other plant species, regardless of their evolutionary or taxonomic rank (Pérez-Massot et al., 2013). Likewise, the favorable genes that were inadvertently ‘left behind’ during the modern breeding process may also be bred back into the cultivated gene-pool, through transgenic interventions, for improving Zn and Fe content in staples (Mhaske, 2019). Additionally, silencing of unfavorable genes (such as regulating phytic acid content) through genome editing may develop cultivars of staple food crops with higher bioavailability of micronutrients (Gyani et al., 2020). A detailed discussion on these aspects is, however, beyond the scope of this review.

The genetic biofortification is often constrained by infrastructure, time, policy regulation, and financial obligation that are unaffordable by the rural and urban poor people who do not have sufficient access to enriched/fortified foods and diversified diets. But once successful, it can be sustainable and environmentally benign on a long-term scale. It is imperative to overcome the complex relationship between Zn and Fe and to breed cultivars for a simultaneous biofortification of staple food crops, and efforts are underway by various country-level and international-level organizations towards it (Gómez-Galera et al., 2010; Andersson, 2017; Senguttuvel et al., 2023). For example, All India Coordinated Rice Improvement Program (AICRIP) in 2013 initiated a biofortification trial that resulted in the release of a few biofortified rice cultivars rich in both Zn and Fe (**Table 5**); such as DRR Dhan 45 (ICAR-Directorate of Rice Research, 2016), CR Dhan 310 (Indian Council of Agricultural Research, 2017). Likewise, Bangladesh has also developed and released several biofortified rice cultivars (e.g. BU Aromatic Hybrid Dhan-1, BU Aromatic Dhan-2, Binadhan 20) to reduce micronutrient malnutrition (http://dhcrop.bsmrau.net). In wheat, such initiatives led to the development of many biofortified cultivars, like WB 02, HPBW 01, HI 8759, MACS 4028 in India (Yadava et al., 2018b) and Kabre in Nepal (Thapa et al., 2022). Biofortified cultivars of other crops include HHB 299 in pearl millet, IPL 220 in lentil, Solapur Lal in pomegranate, with greater accumulation Zn and Fe (Yadava et al., 2018b). Therefore, evidence suggests that Zn and Fe content of food crops can be enhanced as much as 51 mg kg^-1^ and 73 mg kg^-1^ through biofortification programs, respectively.

However, the effectiveness of genetic biofortification and breeding programs for getting nutrient dense crop yields in real world farming systems largely depends on site specific factors including soil nutrient availability, environment and crop management (Mabesa et al., 2013; Zia et al., 2020). Agronomic biofortification can serve as a powerful complementary strategy to ensure stability of these micronutrients in food grains across diverse agro-ecological conditions. For instance, combined soil and foliar Zn application has been reported to improve the performance of a genetically Zn-biofortified wheat variety, with increase in yield, Zn content while also reducing antinutrients (Nayak et al., 2023). This integrated strategy combining genetic and agronomic potential for simultaneous biofortification of Zn and Fe is much needed for getting consistent outcomes under field conditions. In this regard, careful consideration of Zn×Fe interaction is critical to ensure balanced accumulation of these nutrients.

## Conclusion

Agronomic biofortification has certainly emerged as a short-term and cost-effective strategy to curb micronutrient malnutrition on a global scale. A key concern with agronomic Zn biofortification is its uncertain effect on grain Fe content, raising the risk of exacerbating other micronutrient deficiencies. Addressing this trade-off is essential for simultaneous biofortification, without compromising yield or other agronomic traits. We recommend the following interventions to overcome this trade-off, at least from agronomic point of view: (i) maintaining a balance in the phyto-availability of Zn and Fe in soil as when the balance is distorted it underpins the possibility of antagonism at the root level entry, (ii) a conjoint application through soil, foliar, seed priming or a combination, and, (iii) focused research on tailoring Zn and Fe application concurring with higher activity of metal-carriers for simultaneous biofortification. Alternatively, simultaneous accumulation of Zn and Fe in staple food crops can also be achieved through genetic manipulation of metal homeostasis genes. A deeper comprehension of the complexities involving the Zn × Fe interaction, mechanistic studies to understand transporter behavior and nutrient signaling pathways, which ultimately regulate their loading in sink organs, is necessitated for the success of agronomic Zn biofortification programs.
